# The Mycotoxin Patulin Inhibits the Mitochondrial Carnitine/Acylcarnitine Carrier (SLC25A20) by Interaction with Cys136 Implications for Human Health

**DOI:** 10.3390/ijms24032228

**Published:** 2023-01-23

**Authors:** Nicola Giangregorio, Annamaria Tonazzi, Cosima Damiana Calvano, Ciro Leonardo Pierri, Giovanna Incampo, Tommaso R. I. Cataldi, Cesare Indiveri

**Affiliations:** 1 CNR Institute of Biomembranes, Bioenergetics and Molecular Biotechnologies (IBIOM), Via Amendola 122/O, 70126 Bari, Italy; 2 Department of Chemistry, University of Bari Aldo Moro, Via Orabona 4, 70126 Bari, Italy; 3 Department of Pharmacy-Pharmaceutical Sciences, University of Bari Aldo Moro, Via Orabona 4, 70126 Bari, Italy; 4 Department of Bioscience, Biotechnology and Environment, University of Bari, 70126 Bari, Italy; 5 Department DiBEST (Biologia, Ecologia, Scienze della Terra) Unit of Biochemistry and Molecular Biotechnology, University of Calabria, Via Bucci 4C, Arcavacata di Rende, 87036 Cosenza, Italy

**Keywords:** mitochondria, carnitine carrier, patulin, cysteines, inhibition

## Abstract

The effect of mycotoxin patulin (4-hydroxy-4H-furo [3,2c] pyran-2 [6H] -one) on the mitochondrial carnitine/acylcarnitine carrier (CAC, SLC25A20) was investigated. Transport function was measured as [^3^H]-carnitine_ex_/carnitine_in_ antiport in proteoliposomes reconstituted with the native protein extracted from rat liver mitochondria or with the recombinant CAC over-expressed in *E. coli*. Patulin (PAT) inhibited both the mitochondrial native and recombinant transporters. The inhibition was not reversed by physiological and sulfhydryl-reducing reagents, such as glutathione (GSH) or dithioerythritol (DTE). The IC_50_ derived from the dose–response analysis indicated that PAT inhibition was in the range of 50 µM both on the native and on rat and human recombinant protein. The kinetics process revealed a competitive type of inhibition. A substrate protection experiment confirmed that the interaction of PAT with the protein occurred within a protein region, including the substrate-binding area. The mechanism of inhibition was identified using the site-directed mutagenesis of CAC. No inhibition was observed on Cys mutants in which only the C136 residue was mutated. Mass spectrometry studies and in silico molecular modeling analysis corroborated the outcomes derived from the biochemical assays.

## 1. Introduction

PAT (4-hydroxy-4H-furo [3,2c] pyran-2 [6H] -one) is a mycotoxin produced by molds, such as *Penicillium* and *Aspergillus*. It has been found in fruit (particularly in apples and red globe grapes) and vegetable products, but also as a contaminant in derivatives, such as fruit juices or animal feed. Several studies have demonstrated the potentially toxic effects of PAT on human and animal health. PAT can withstand high temperatures during processing events and storage, so the possibility for life-long exposure to PAT and the increase of its intracellular dose due to successive ingestions can produce serious complications to vital organs, such as the liver, kidney, intestine, immune system, and nervous system in experimental animal models; it can also lead to carcinogenesis due to chromosomal DNA damages [1,2,3,4].

Although the PAT content in apples and apple products established by WHO is within acceptable limits (50 μg/L), there are some countries in which the levels detected in fruit and derivatives represent a high risk for human health [5,6,7]. The major danger is caused by prolonged exposure to low doses of PAT which may lead to chronic toxicity and thus irreversible effects. There are some categories of more vulnerable people, such as children under 12 [8] and fetuses [9]. Indeed, PAT influences their development.

PAT is an unsaturated heterocyclic lactone with electrophilic properties, which can undergo nucleophilic attack from thiol (SH) groups of the cysteine residues [10], causing the inhibition of many enzymes [11]. PAT was shown to affect the activity of enzymes involved in the glycolytic pathway or gluconeogenesis [12] or the protein tyrosine phosphatase (PTP) [13]. In this latter case, the molecular mechanism of inhibition is due to the irreversible covalent binding of PAT on the Cys 215, located in the active site of PTP, and essential for enzymatic activity [14].

Earlier, we demonstrated that the mitochondrial carnitine/acylcarnitine carrier (CAC, SLC25A20), has specific cysteine residues that target inorganic (Hg^2+^, Cu^2+^) chemical species, which impair the mitochondrial carnitine transport in vitro, in vivo, and in the animal zebra fish model [15,16].

Besides, we found that some pharmacological compounds, such as β-lactam antibiotics, are toxic to the transporter functionality [17]. CAC is pivotal for the transport of acyl groups as acylcarnitines into the mitochondrial matrix, where the acyl moieties are catabolized by the β-oxidation pathway [18]. CAC’s transport activity is modulated by several physiological effectors, such as GSH, H_2_S, NO, and CO [19,20,21,22], as well as by several xenobiotics and drugs (e.g., MeHg, β-lactam antibiotics, omeprazole, dantrolene) [15,17,23,24] acting on one or two specific Cys residues, namely C136 and C155, identified by site-directed mutagenesis.

In this paper, by using multiple experimental approaches, we have investigated the CAC’s sensitivity to PAT by studying its effects both on the mitochondrial and recombinant proteins. We identified the cysteine residue involved in the binding with PAT by site-directed mutagenesis, MS, and in silico molecular modelling analysis.

## 2. Results

### 2.1. Effect of Patulin on the Mitochondrial CAC

**Figure 1:**
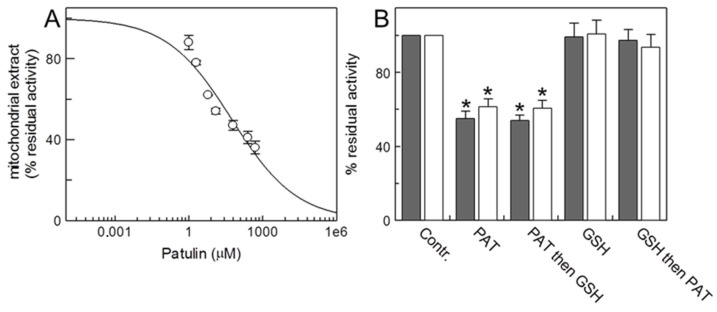
*Effect of Patulin on the native carnitine/acylcarnitine carrier (CAC).* (**A**) Dose-response analysis of proteoliposomes reconstituted with the CAC extracted from rat liver mitochondria (0.3 mg of total proteins) and incubated with PAT at the indicated concentrations for 30 min, before measuring the transport activity by adding 0.1 mM [^3^H] carnitine for 10 min as described in Materials and Methods. The percentage of residual activity compared to control, without PAT treatment, is reported. The values are the means ± SD from three independent experiments. (**B**) Rat liver mitochondria (5 mg/100 μL) were incubated or not (control) for 60 min, with Patulin (60 μM) or GSH (5 mM) as indicated. Upon treatment, the unreacted drug was removed by washing the mitochondria in PBS 1X and then 0.3 mg mitochondria were solubilized by 3% TX-100, 10 mM PIPES pH 7.0 and reconstituted in proteoliposomes for transport assay as indicated in (**A**) and Materials and Methods. Black and white bars indicate without or with the addition of 5 mM dithioerythritol (DTE) 1 min before the transport assay to aliquots of each sample. The data are expressed as the percentage of the controls (no addition of PAT) and are means ± SD from three independent experiments; significantly different from the respective control as estimated by Student’s *t*-test (* *p* < 0.01).

For studying the effect of PAT on the native mitochondrial CAC, a dose–response analysis on the rat liver-extracted mitochondria was performed. Aliquots of the mitochondrial extract (0.3 mg of total proteins) were reconstituted in liposomes and incubated for 30 min with several concentrations of PAT before starting the transport assay as [^3^H]-carnitine/carnitine antiport (see Materials and Methods). As shown in Figure 1A, the curve suggests a CAC inhibition by PAT with a calculated IC_50_ equal to 55 ± 6.0 µM.

The effect of PAT on the CAC at 60 µM concentration was also detected in intact rat liver mitochondria. Upon treatment (60 min), the mitochondria were solubilized in Triton X-100 and 0.3 mg of mitochondrial extract were reconstituted in proteoliposomes for testing the transport activity of CAC (see above). Figure 1B shows that PAT inhibits approximately 50% of the CAC transport activity in the mitochondria compared to the control. Aliquots of intact mitochondria incubated with PAT were treated also with reduced GSH to verify whether this physiological SH-reducing agent was able to reverse the mycotoxin-protein interactions.

The addition of GSH, after PAT treatment, did not lead to a recovery of the transport activity, suggesting an irreversible covalent interaction of the mycotoxin occurring with the protein’s cysteine(s). No reverse binding was also observed when DTE, a strong chemical SH-reducing agent [25], was added to the proteoliposomes during the transport assay (Figure 1B white columns). However, GSH added to mitochondria before PAT incubation prevented its inhibition, whereas the addition of GSH alone produced only a moderate increase in the transport activity compared to the control (Figure 1B).

### 2.2. Effect of Patulin on the Recombinant CAC WT and Site-Directed Cys Mutants

**Figure 2:**
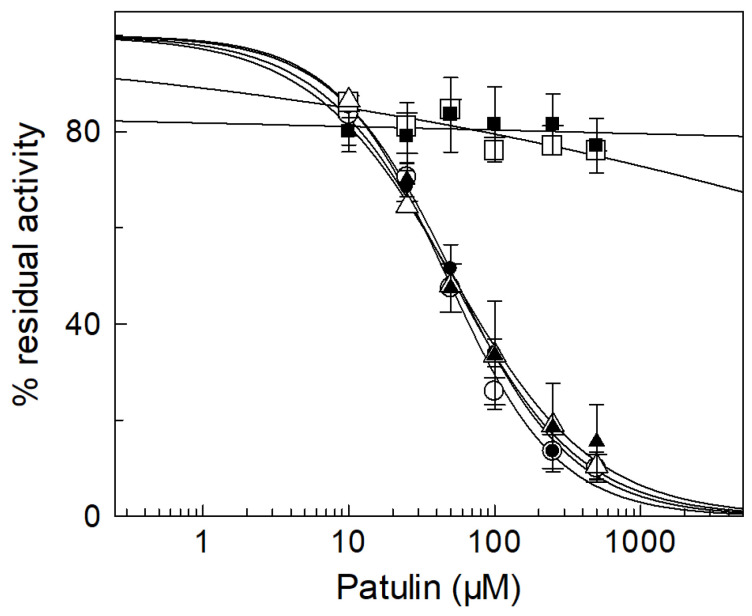
*Dose-response analysis of the PAT inhibition of the CAC recombinant proteins.* Transport activity of the reconstituted rat (○) and human (●) WT, C136S (□), C23V/C58V/C89S/C136V/C283S (■), C155S (Δ) and C23V/C58V/C89S/C155V/C283S (▲) was measured by adding 0.1 mM [^3^H]-carnitine after PAT treatment at the indicated concentrations for 30 min. The data are expressed as a percentage of the controls (no PAT addition) and are means ± SD of three independent experiments.

To shed light on the interaction between PAT and CAC and verify if specific Cys residues were involved, transport experiments on the recombinant rat and human WT CAC and CAC’s rat Cys mutants were performed. As in the case of the native protein, transport activities were measured as [^3^H]-carnitine/carnitine antiport in proteoliposomes. From the dose-response analysis of rat and human CAC WT inhibition, IC_50_ of 47 ± 9.2 µM and 50 ± 7.8 μM were respectively obtained (Figure 2). The PAT inhibition of the WT recombinant protein was only slightly greater compared to the native protein, although compounds and other molecular systems that affect the mycotoxin binding to the native CAC were absent (see above). For identifying the residues involved in the PAT interaction, site-directed Cys mutants were tested for inhibition (Figure 2). The sensitivity to PAT was strongly decreased in the recombinant proteins lacking C136, such as the mutants C136S and C23V/C58V/C89S/C136V/C283S (containing only C155). The choice of this last mutant was due to the proximity of C155 to C136 during some steps of the substrate translocation mechanism [25,26]. However, in both mutants, it was not possible to detect the IC_50_ value, implying that the interactions of PAT with C136 are determining factors for CAC’s inhibition. To confirm the main role played by C136 in the carrier PAT-induced impairment, the single mutant C155S and the mutant containing only C136 residue (C23V/C58V/C89S/C155V/C283S) were examined (Figure 2). A strong inhibition, with IC_50_ values comparable to the WT, was observed. As previously demonstrated [16,19,20], these data indicate that, although Cys155 plays an important role in CAC inactivation in the presence of some physiological or chemical compounds, only the C136 residue of the carrier is reactive towards PAT, causing CAC inhibition. Finally, no effect of the mycotoxin was observed on the C-less mutant (C23V/C58V/C89S/C136V/C155V/C283S), definitively excluding the participation of other residues in the interaction with PAT.

**Figure 3:**
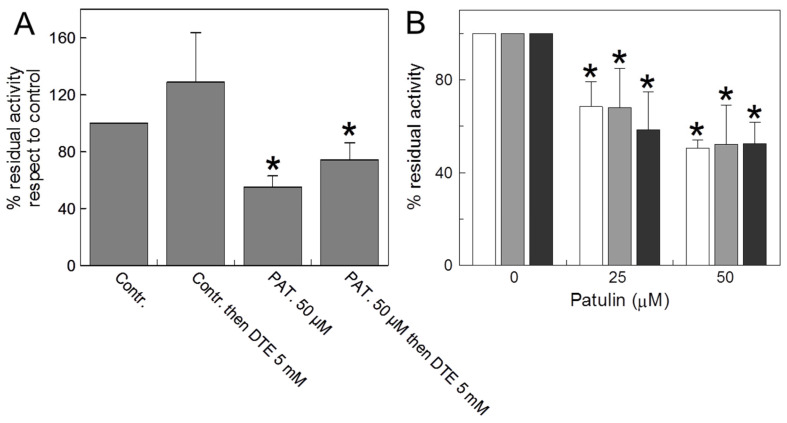
(**A**) *Reversal of PAT inhibition on recombinant protein by DTE.* The carnitine homo-exchange transport activity was measured, as described in Materials and Methods, adding 0.1 mM [^3^H] carnitine to proteoliposomes reconstituted with the recombinant WT CAC containing 15 mM internal carnitine in the absence or presence of 50 μM PAT, added 30 min before the labelled substrate. 5 mM dithioerythritol (DTE) was added 1 min before the transport assay in a control and PAT treated aliquot, respectively. The transport was stopped after 30 min by 1.5 mM NEM. (**B**) *Time-dependent reactivity of the same PAT solution on the recombinant WT protein*. Proteoliposomes reconstituted with the WT CAC were incubated by the same PAT solution (25 and 50 µM) after 0.5 h, 24 h and 72 h (white, grey and black bars, respectively) and then the transport activity was measured by adding 0.1 mM [^3^H]-carnitine and stopped after 30 min as described in Materials and Methods. The data of both panels are expressed as a percentage of the residual activity compared to the control, without PAT treatment, and are means ± SD from three independent experiments; significantly different from the respective control as estimated by Student’s *t*-test (* *p* < 0.01).

Figure 3A reports the interaction of PAT with the reconstituted WT protein. The inhibition observed after the pretreatment of proteoliposomes with the mycotoxin was not reversed by DTE, confirming the formation of an irreversible covalent bond between CAC and PAT also with the purified recombinant protein (see above Figure 1B). For evaluating the stability of the drug following prolonged storage, we measured the inhibition of the transport activity of WT adding to the samples, at various time, the same PAT solution stored at room temperature (r.t.). As shown in Figure 3B, the reactivity of 25 and 50 µM PAT towards CAC at both 24 and 72 h does not change, indicating that PAT is chemically stable and forms stable interactions with the protein during the incubation time.

**Figure 4:**
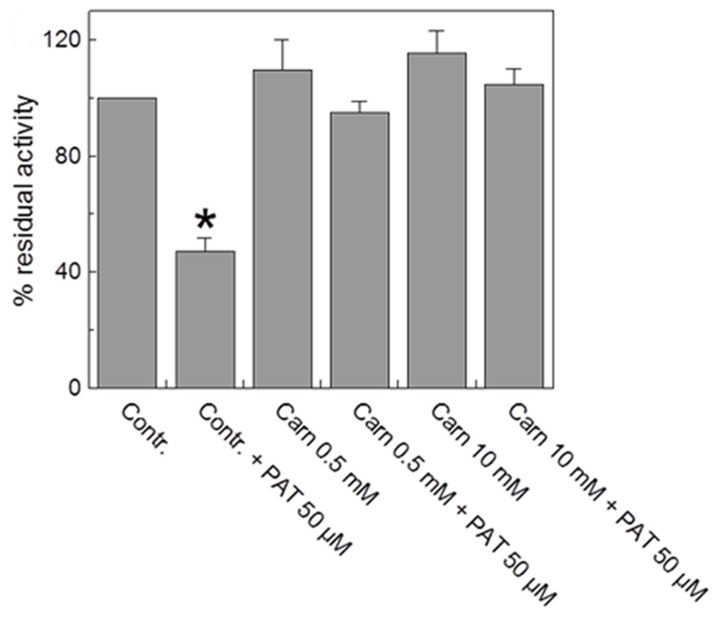
*Substrate protection on the PAT inhibition of CAC*. Carnitine at the indicated concentrations was added to proteoliposomes, and then the mixture was treated for 30 min with (grey bars) or without (white bars) the addition of 50 μM PAT. Then, the unreacted compound was removed by passing the proteoliposomes through Sephadex G-75 columns (see Materials and Methods). Transport was started by adding 0.1 mM [^3^H]-carnitine to the proteoliposomes and stopped by 1.5 mM NEM after 10 min. The residual activity is reported with respect to the control without the addition of PAT. The data are the means ± SD from three different experiments; significantly different from the respective control as estimated by the Student’s *t*-test (* *p* < 0.01).

To investigate PAT interactions with residues of the substrate binding area, a protection experiment was performed in which carnitine was added to the CAC at different concentrations before the inhibitor. As shown in Figure 4, the addition of carnitine both at 0.5 and 10 mM, partially prevented PAT inhibition, most likely because the target of the mycotoxin could be the thiol group of C136 located on the bottom of the substrate binding area, thus suggesting a steric hindrance to the substrate passage through the protein water-filled cavity. To reject nonspecific effects independent of mycotoxin, carnitine was added to some samples in the absence of PAT, before the transport activity was started. No variations were observed (Figure 4).

### 2.3. Multiple Sequence Alignment and 3D Molecular Modelling of RnCAC (Rattus norvegicus CAC)

**Figure 5:**
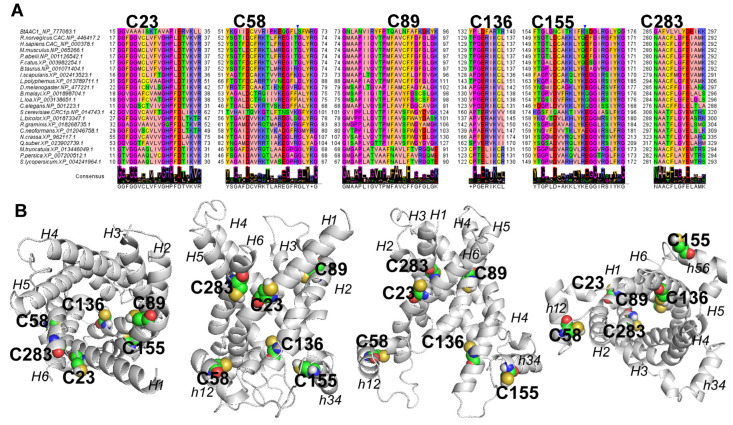
Multiple sequence alignment and 3D comparative modelling of CAC. An extract of the multiple sequence alignment of CAC protein closest homologs, sampled by BlastP, through Mammalia, Arthropoda, Nematoda, Fungi and Plants is reported in panel (**A**). The reported CAC protein fragments host the investigated cysteine residues. The proposed alignment was obtained by using the zappo colour style of Jalview. Bold labels indicate the location of the cysteine residues, investigated by site-directed mutagenesis and transport assays in this work. In panel (**B**), the 3D comparative models of CAC protein in c-conformation (starting from the left, top view and lateral view) and m-conformation (lateral view and bottom view) are reported in a grey cartoon. Cysteine residues are reported in spheres representation and labelled for comparative purposes. The transmembrane helices H1-H6 parallel to the membrane plane and the short helices, h12, h34, and h56 are indicated by italics labels.

To link PAT inhibition with the conservation and localization of the investigated cysteine residues on the CAC protein, we built a multiple sequence alignment and the 3D comparative model of CAC (Figure 5A). The obtained multiple sequence alignment revealed that the investigated cysteine residues appear strongly conserved in mammalian CAC orthologous sequences. In order to gain new insights about the role played by the investigated cysteine residues we performed a structural analysis by building the all-atom 3D comparative protein models of the WT CAC in the cytosolic conformation (c-conformation or c-state) and matrix conformation (m-conformation or m-state).

The resulting 3D comparative models (Figure 5B) of the CAC in c-conformation and in m-conformation consist of six helices (H1-H6) perpendicular to the membrane plane and three short helices (h12, h34, h56) parallel to the membrane plane. The 3D comparative model of CAC in the cytosolic conformation (c-conformation), open towards the intermembrane space, was based on the crystallized *Bos taurus* AAC1 (1okc.pdb, [27]) that was used as a protein template to guide the 3D comparative modeling session. The RMSD between the coordinates of the atoms of the backbone of the obtained CAC 3D comparative model in c-conformation and the corresponding atoms of the crystallized BtAAC1 was equal to 0.362 Å. Residues D32, K35 on transmembrane helix 1 (H1), E132, K135 on transmembrane helix 3 (H3), and D231, K234 on transmembrane helix 5 (H5), located at the C-terminal of odd helices, close to the matrix face, are involved in the formation of inter helix ion pairs, forming the matrix gate (m-gate) in the CAC 3D model in c-conformation. The m-gate closes the carrier on the matrix face [28,29].

The 3D comparative model of CAC in matrix conformation (m-conformation), open towards the matrix was based on the crystallized *T. thermophilus* AAC1 (6cgi.pdb, [30]) that was used as a protein template to guide the 3D comparative modelling session. The RMSD between the coordinates of the atoms of the backbone of the obtained CAC 3D comparative model in m-conformation and the corresponding atoms of the crystallized TtAAC1 was equal to 0.221 Å. Residues G95, K97 on transmembrane helix 2 (H2), E191, K194 on transmembrane helix 4 (H4), and E298, M291 on transmembrane helix 6 (H6), located at the c-terminal of even transmembrane helices, close to the intermembrane face, participate in the formation of inter helix ion pairs, forming the cytosolic gate (c-gate), in the CAC 3D model in m-conformation. The c-gate closes the carrier on the intermembrane space [28,31].

By projecting the investigated cysteine residues on the 3D structure, a comparative model of RnCAC (Figure 5B), both in c-conformation and in m-conformation, it was observed that the investigated cysteine residues C23 (H1, between residues of PG-level 1 and residues of the substrate binding site) and C283 (H6, between the substrate binding site and the cytosolic gate) protrude towards the membrane in the c-conformation. Conversely, in the m-conformation, only C23 faces the membrane, whereas C283 protrudes towards the carrier cavity. C89 (H2, located between the substrate binding site and the cytosolic gate) and C136 (H3, located at the level of m-gate residues) protrude towards the substrate translocation cavity in both the c-conformation and m-conformation. C58 and C155 are part of the proposed mitochondrial carrier (MC) regulatory site [26,31] at the level of the small helices h12 and h34 parallel to the membrane plane and protrude towards the central axis of the carrier cavity but locate approximately behind transmembrane H1 and H3 in both the c-conformation and m-conformation.

### 2.4. Docking Analysis

**Figure 6:**
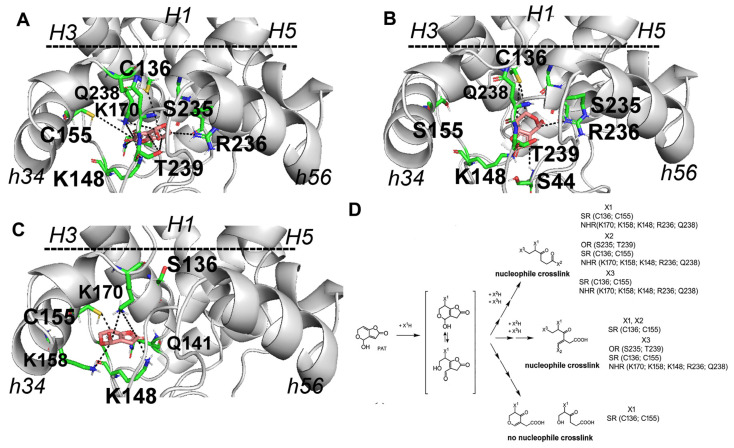
PAT docking analysis and possible interactions between PAT and residues of the matrix gate area, including matrix loop residues and residues of the short helices h12, h34 and h56 perpendicular to the membrane plane. A zoomed view of the matrix gate area residues and residues of the short helices h12, h34 and h56 interacting with PAT (salmon sticks) as obtained by docking analysis on the 3D comparative model of the WT CAC (panel (**A**)), CAC_C155S (panel (**B**)) and CAC_C136S (panel (**C**)). The 3D comparative model of the CAC protein is reported in grey cartoon representation. Protein residues between the m-gate, matrix loops, and short helices h12, h34, h56, within 4 Å from the docked PAT are reported in green sticks and labelled for comparative purposes. The black horizontal dashed bar indicates the position of the m-gate. Dashed lines indicate inter-residues distances below the 5 Å. Possible nucleophilic attacks on PAT are reported in the scheme of panel (**D**). The transmembrane odd helices H1, H3 and H5, and the short helices h34 and h56 are indicated by labels.

In order to monitor what happens at the level of the investigated C136, which is the cysteine making CAC more sensitive to PAT in in vitro transport assays, we performed a dedicated docking analysis in the 3D comparative model of CAC in c-conformation (Figure 6). Our docking analysis showed that PAT establishes strong (in the 4 Å range of distances) interactions with T40, C136, Q141, K148, K170, C155, S235, R236, Q238, and T239 in the CAC wild type protein, located between the matrix gate and residues of the short helices h12 and h34 (Figure 6A). Notably, the docking of PAT in the CAC mutant hosting only C136 showed a few differences in the amino acids involved in the PAT binding pocket, here consisting of residues T40, S44, C136, K148, S235, R236, Q238, and T239 mainly located at the level of residues of the matrix gate area (Figure 6B). Conversely, during the docking of PAT in the 3D CAC mutant hosting only C155, a different set of interactions protruding towards the CAC matrix loop at the level of the short helix h34 was observed and included residues Q141, K148, C155, K158, and K170 (Figure 6C).

### 2.5. Mass Spectrometry Analysis

The interaction between C136 and PAT was further investigated by mass spectrometry (MS). The gel band at approximately 30 kDa, which is the putative molecular weight (MW) of CAC, was excised for both control and sample reacted with 100 µM PAT (see Materials and Methods). In-gel tryptic digestion was accomplished on each piece and the resulting mixture of peptides was examined by MALDI-MS. Supplementary Figure S1 shows the mass spectra of both the control and PAT-treated samples, respectively, in panels A and B, while Table 1 lists the experimental and theoretical *m*/*z* values of those identified peptides. In both cases, the peptide mass fingerprinting (PMF) allowed us to recognize the mitochondrial CAC protein with good sequence coverage.

A preliminary assessment of the MALDI MS spectra showed no significant differences between the investigated samples, most likely because the signals coming from the modified peptides are suppressed from native ones, which are both more abundant and easily ionizable. However, we focused on the putative modifications of cysteines in the CAC sequence and in particular on the tryptic peptide containing the C136, as annotated in Table 1. While the expected peak signal of the following sequence 136–146, i.e., CLLQIQASSGK at *m*/*z* 1147.57, was not present in the PAT-treated sample (Supplementary Figure S2), a peak signal at *m*/*z* 2456.23 was observed (Supplementary Figure S3). This last was assigned to a greater number of amino acid residues, including the protein chain sequence 136–15 and matching the peptide **C**LLQIQASSG*K*N*K*YSGTLD**C**A*K*K with three missed cleavages.

**Figure 7:**
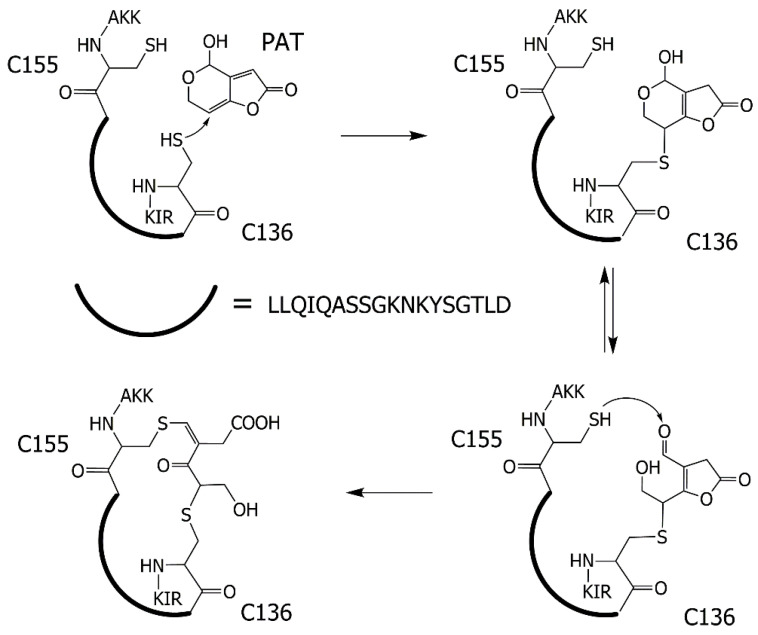
A possible pathway of the PAT reaction with the amino acid sequence **C**LLQIQASSG*K*NKYSGTLD**C**A*K*K. A final cyclic peptide with an intramolecular bond between C136, PAT and C155 was hypothesized. For residues preceding C136 (133-RIK-135) and following C155 (156-AKK-158), see also Figure 5.

In order to check for the presence of a group of PAT-mediated crosslinked residues from different CAC protein secondary structure elements, as a consequence of a multiple nucleophilic attack on PAT by CAC residues, involving C136 and C155, we hypothesized the existence of a cyclic peptide with a mass shift of +154 Da (Figure 7). Interestingly, an accurate examination of both spectra in Supplementary Figure S1 permitted us to discover the occurrence of a very small peak at *m*/*z* 2610.23 in the PAT-treated sample, whereas it was not detected in the control one (see Supplementary Figure S4).

**Figure 8:**
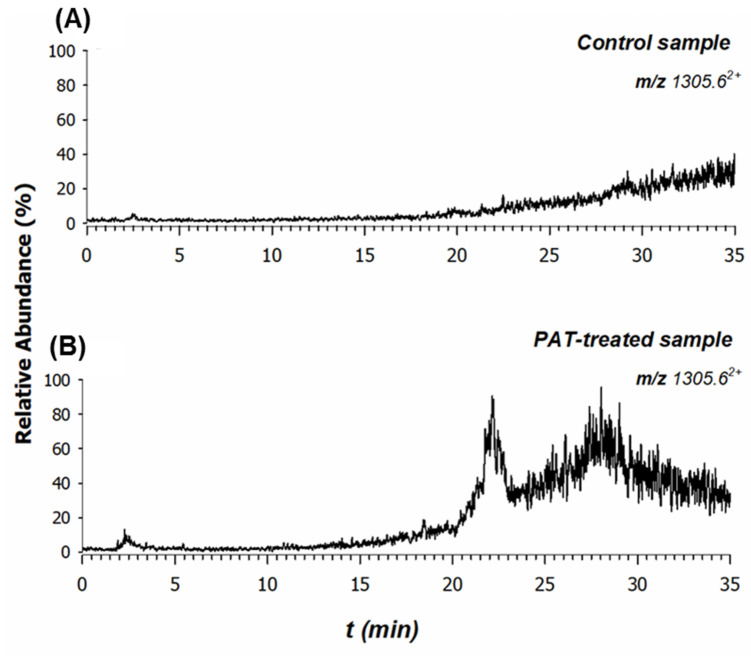
Extracted ion current (XIC) chromatograms at *m*/*z* 1305.6^2+^ of the modified peptide searched in the tryptic digest of (**A**) control and (**B**) treated samples.

Three different PAT-treated samples were prepared and investigated by MALDI MS. In all cases, the peak signal at *m*/*z* 2610.23 exhibited a very low signal-to-noise ratio and no reliable tandem MS spectra were acquired. An attempt was carried out by analyzing both control and PAT-treated samples in reversed-phase liquid chromatography coupled with electrospray ionization and MS (RPLC-ESI-MS). As an example, the extracted ion current (XIC) chromatogram of the bi-charged ion at *m*/*z* 1305.6^2+^ is reported in Figure 8 for control (A) and PAT-treated (B) samples. As expected, the absolute intensity of this peptide is very low, but its occurrence can be appreciated as it occurs at ca. 22 min only in the PAT-treated sample, confirming the suggested formation of a cyclic peptide prompted by PAT. Since the proteomics database search did not allow the selection of PAT-induced modifications, a manual spectrum validation was mandatory to explain product ions associated with the modified peptides.

**Figure 9:**
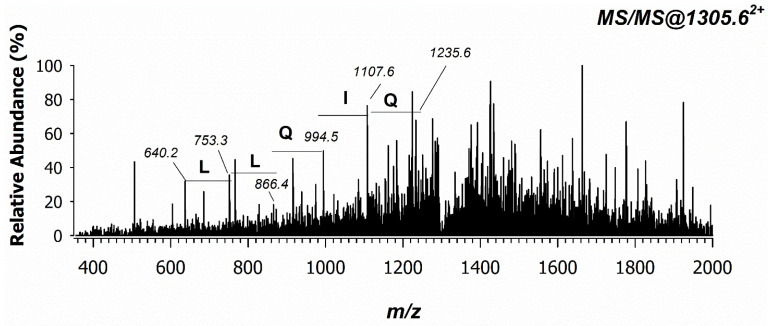
ESI MS/MS mass spectrum of the doubly charged precursor ion at *m*/*z* 1305.6^2+^ observed in the PAT-treated sample and generated from the cyclic peptide formed by intramolecular bonds between PAT and two -SH groups of cysteines 136 and 155 of the sequence **C**LLQIQASSGKNKYSGTLD**C**AKK.

Although the tandem MS spectrum of the doubly charged precursor ion at *m*/*z* 1305.6^2+^ was found to be very noisy (Figure 8), some peak signals were recognized and assigned. In contrast to the direct sequence (DS) ions derived from typical fragmentation pathways by CID-MS/MS experiments, cyclic peptides give rise to unusual product ions generated by nondirect sequence (NDS) ions. These product ions are based on the peptide scrambling sequence as a result of spontaneous recyclization events [32]. Yet, some product ions of the peptide sequence were identified, as reported in Figure 9.

## 3. Discussion

In this report, we have investigated the inhibition of the transport activity of the mitochondrial CAC by PAT. This mycotoxin inhibits the carrier on both the native (isolated rat liver mitochondria) and the human and rat recombinant wild-type proteins. In intact rat mitochondria, the IC_50_ value was 60 µM, slightly higher than IC_50_ of the WT protein reconstituted as proteoliposomes, however a dose in the nontoxic range for cell viability [33]. Patulin is a stable mycotoxin (see the introduction and Figure 3B), so it is likely that the in vivo IC_50_ concentration may be achieved after long exposure (bioaccumulation) both in the cellular and mitochondrial compartment. The consequences of the prolonged ingestion of food contaminated with PAT lead to chronic toxic health effects [7]. It is known that PAT enters into the cells via the vesicular transport mechanism; since it increases the expression of proteins related to endocytosis, its cell accumulation is favored [34]. We previously demonstrated, by experiments conducted on zebrafish (*Danio rerio*), that the toxic bioaccumulation of mercury (biomagnification) also inhibits the mitochondrial CAC, by a covalent reversible interaction with the carrier [15]. PAT in vivo biomagnification could be more toxic and dangerous for CAC, compared to mercury, because neither reducing chemical reagents such as DTE nor the physiological reducing GSH, which we have shown to be important to keep the carrier in its active state [19], were able to reverse the PAT covalent bond with thiol groups (Figure 1B and Figure 3A).

The cysteine residues of CAC involved in the PAT binding interactions, resulting in the inhibition of CAC transport activity, were identified by site-directed mutagenesis. We mutated the two cysteine residues (C136 and C155) that we also previously indicated to be specific nucleophilic sites for several chemical and physiological compounds [15,16,19,20,21,22]. Dose–response analysis of several Cys mutants highlights that PAT inhibits the carrier by binding all the mutants containing only the C136 residue and recording IC_50_ values such as WT (Figure 2). Interestingly, mutants lacking C136 but containing the C155 residue, the other reactive cysteine located close to C136 during the substrate translocation [18,26], do not inhibit the transporter following putative interactions with PAT. Indeed, no IC50 value could be estimated with the above-cited mutants (Figure 2).

All the conclusions reported above were also supported by in silico analysis. We generated 3D models of CAC protein in cytosolic conformation, where C136 and C155 are closer (approximately 8 Å, compared to what was observed in the m-conformation where their distance is greater than 18 Å), and investigated possible crosslinks triggered by PAT (docking analysis) following nucleophilic attack due to those two cited cysteines as well as other basic amino acids [10,35,36]. In the presence of both cysteine residues (WT), the docking analysis allowed to highlight the possible formation of a network of interactions between PAT and residues T40, C136, Q141, K148, K170, C155, S235, R236, Q238, and T239 located between the matrix gate and residues of the short helices h12 and h34 (Figure 6A). In addition, the replacement of C155 with a serine (Figure 6B) or valine did not produce a significant variation of amino acids involved in the PAT binding pocket, here consisting of residues T40, S44, C136, K148, S235, R236 Q238, and T239 (Figure 6B). Conversely, when PAT was docked in the CAC mutant hosting only C155, as cysteine residue, a different set of interactions was observed, including residues Q141, K148, C155, K158, and K170. The main difference observed by docking analysis among the three different highlighted binding pockets concerns the involvement of residues S235, R236, Q238, and T239, located on the third CAC repeat, with specific reference to the end of transmembrane H5, at the level of the m-gate area in presence of C136. It is proposed that the binding interactions between the investigated C136, together with the basic residues highlighted by docking analysis, could be responsible for the formation of inter-repeat crosslinked CAC-PAT protein-ligand complexes, according to what is expected from the major reaction of PAT with nucleophilic groups from cysteine and lysine residues [10,35,36]. It is also suggested that the cross-link between PAT and residues of the three repeats of CAC, including C136, prevents conformational changes and thus inhibits carnitine translocation. In the absence of C136, despite the presence of C155, it appears that PAT is no longer able to crosslink residues of the first two repeats of CAC with residues of the third repeat. Indeed, in this case, PAT binds to a highly mobile loop region of CAC between transmembrane helix 3 and the short helix h34. It can be speculated that the binding of PAT at the highlighted highly mobile loop does not affect/prevent conformational changes, thus allowing carnitine translocation. This last interpretation is supported by the IC_50_ experiments (Figure 2) where the carrier is inhibited by PAT only in the mutants containing C136 residue.

Mass spectrometry analysis was then used to identify the putative covalent modifications of CAC by PAT binding justifying the formation of a PAT-mediated crosslink between C136 and C155 in support of the above reported functional results. In this regard, a paper by Pfeiffer et al. [37] suggested that up to three thiol groups of reduced GSH molecules can react with one PAT molecule. The same authors also proposed that the initial reaction of PAT with one thiol of GSH leads to an adduct, which is even more reactive towards thiols and amino groups than PAT itself [10].

Searching for a product resulting from a multiple nucleophilic attack, a peak signal at *m*/*z* 2456.23 (see Table 1) was highlighted, not detected in the control, but detected in the PAT-treated samples, matching to a peptide including the residues C136 and C155. It was assumed a cross-link between PAT and the thiol groups of C136 and C155 with the generation of a long cyclic peptide responsible for the cited peak at *m*/*z* 2456.23. The formation of this longer peptide may be due to the decreased proneness of CAC region hosting the investigated C136-C155 loop to protease digestion, as a result of conformational changes and lower enzyme accessibility of CAC in the tight c-conformation strengthened by the C136-PAT-C155 crosslink. Conceivably, these preliminary results deserve further investigations on enriched PAT-treated samples, perhaps using better-tailored strategies of sample pretreatment and enrichment. Importantly, C136 represents the first residue involved in a nucleophilic attack on PAT, as proposed in the reaction mechanism of Figure 7, since it is located in the bottom of the water-filled cavity of CAC, where the substrate binds (see Figure 4 and the docking analysis of Figure 6), inducing substrate protection in the protein. On this concern, the cyclic peptide observed by mass spectrometry is compatible with a PAT-induced cyclic peptide consisting of the C136–C155 loop residues. Conversely, in the absence of C136 or C155, PAT can undergo both nucleophilic attacks with and without the formation of crosslinked peptides, according to what is suggested by Figure 6D (see also [35]).

By the interaction of metals such as mercury with GSH or in pathological conditions, such as in ischemia-reperfusion injury (see below) and other pathologies, such as hypertension [38,39], ROS scavenging is reduced due to the significantly lower GSH/GSSG ratio, and the antioxidant mechanisms become inadequate. Owing to oxidative stress, the bioaccumulation of PAT can induce the further depletion of mitochondrial glutathione, given its high affinity for reduced GSH [34,40], thus generating an oxidative environment and the later inhibition of CAC’s transport activity. Our data confirm the interaction between GSH and PAT and highlight how mycotoxin affects CAC transport activity under different redox states. Indeed, the addition of PAT outside the intact mitochondria (oxidized system) before GSH is not able to prevent the binding of the mycotoxin with the CAC and the consequent inhibition of the protein (Figure 1B). Note that the preliminary addition of GSH (reduced system) leads to CAC protection from the mycotoxin binding (Figure 1B).

As we have previously demonstrated, CAC is extremely sensitive to redox changes in response to gasotransmitters and/or other reactive compounds [15,16,19,20,21,22]. For instance, we proposed that H_2_S [20] would favor cardiovascular protection under the condition of oxidative stress (ischemia-reperfusion injury), suppressing the β-oxidation following CAC inhibition [41,42]. Likewise, PAT’s binding to CAC prevents the β-oxidation of fatty acids in the mitochondria, with the consequent reduction of OXPHOS and ROS production. Hence, the toxic effect of PAT on the carrier may mitigate the severe oxidative stress occurring in cardiovascular pathologies [41,42]. Besides, PAT may reduce cell apoptosis, as demonstrated in several papers on HEK293 or HepG2 cells [43,44,45,46]. Finally, the reactivity of the mycotoxin towards CAC, especially C136, suggests that PAT can be considered a xenobiotic redox regulator of the carrier, such as the physiological compounds GSH, H_2_S, NO, and CO [19,20,21,22].

## 4. Materials and Methods

### 4.1. Chemicals

L-[methyl-^3^H]-carnitine from Scopus Research BV Costerweg, Sephadex G-75, egg-yolk phospholipids (l- α phosphatidylcholine from fresh turkey egg yolk), PIPES, Triton X-100, cardiolipin, L-carnitine, N-ethylmaleimide (NEM), Patulin (PAT), water, acetonitrile, ammonium bicarbonate, 4-chloro-α-cyanocinnamic acid (CClCA), and formic acid for MS analysis were purchased from Sigma-Aldrich (Milan, Italy). All solvents used were LC–MS grade. The calibrating solution of the orbital trap MS instrument (q-Exactive) was purchased from Thermo Scientific (Waltham, MA, USA) while a mixture of ACTH 18–39 fragment, angiotensin I, and renin obtained from Sigma-Aldrich was used for MALDI MS calibration. All other reagents were of analytical grade.

### 4.2. Overexpression of the WT and Mutant CACs

The pMW7-WT rat or human CAC recombinant plasmids were used for over-expressing the rat and human CAC, as previously described [28,47]. To introduce the mutations on the rat CAC recombinant plasmid, the overlap extension method was used as described, introducing the NdeI and HindIII restriction sites [48]. The PCR products were purified using the QIAEX II Gel Extraction Kit (QIAGEN, Hilden, Germany), digested with NdeI and HindIII, and then ligated into the pMW7. All mutations were verified by DNA sequencing. The resulting plasmid constructs were transformed into *E. coli* C0214. Overexpression, inclusion body fraction preparation, and purification of the CAC proteins were performed as previously described [49].

### 4.3. Reconstitution of Mitochondrial and Recombinant Protein of CAC in Proteoliposomes

The recombinant CAC proteins were reconstituted in proteoliposomes as previously described [49]. Rat liver mitochondria after purification, using the standard procedure of cell disruption and differential centrifugation [50] were solubilized with 3% Triton X-100 in a PIPES buffer (10 mM) at pH 7.0. Herein, the mitochondrial extract (0.3 mg) were reconstituted in the liposomes as above described for the mitochondrial CAC recombinant protein, in the absence of reducing agents. The concentration of intraliposomal carnitine was 15 mM in all samples.

### 4.4. Transport Assay in Proteoliposomes

For the transport assay, 550 μL of proteoliposomes were passed through a Sephadex G-75 column in order to remove the external substrate. Six hundred μL of the turbid eluate were collected, divided into samples of 100 μL each, and used for transport measurement by the inhibitor-stop method [51]. Transport was started as homoexchange by adding 0.1 mM [^3^H]-carnitine to proteoliposomes containing 15 mM carnitine and, at the indicated time interval, stopped by 1.5 mM NEM (N-ethylmaleimide). The inhibitor was added together with the labelled substrate at time zero in the control samples. After finishing the transport reaction, the external radiolabel substrate was removed by gel filtration chromatography (Sephadex G-75), and the intraliposomal radioactivity was measured by liquid scintillation [51]. Transport was measured at 25 °C. The experimental values were corrected by subtracting the controls. Transport rates were measured in 10 min, i.e., within the initial linear range of the time course.

### 4.5. MC Protein Multiple Sequence Alignment

The *Rattus norvegicus* CAC (RnCAC) protein sequence (NP_446417.2) was used as a query sequence for sampling the CAC protein closest homologs through metazoa, fungi and plants from the NCBI (refseq_protein). The sampled sequences were multi-aligned with CLUSTALW (http://www.clustal.org/clustal2/, accessed on 20 November 2022) according to our previously defined criteria [31,52]. The Bos taurus protein sequence BtAAC1 (RefSeq ID NP_777083) was used as a reference sequence in residue numbering and structural annotation. The resulting alignment was manually refined using the secondary BtAAC1 structure to introduce gaps in the alignment using Jalview as previously described [31,52] for evaluating the conservation of the investigated cysteine residues.

### 4.6. 3D Protein Structure and Comparative Modeling of RnCAC Cys Mutants

The comparative 3D structural models of the SLC25A20_CAC protein in c-conformation and m-conformation were prepared by using SwissPDBViewer (SPDBV, http://spdbv.vital-it.ch/; accessed on 20 November 2022). The crystallized structures of BtAAC1 or TtAAC1 were used as protein templates for guiding the 3D comparative modelling session of CAC in c-conformation or m-conformation, respectively. The structure of BtAAC1 has the highest resolution (2.20 Å) among the available AAC crystallized structures in the Protein Data Bank and it shows the highest percentage of identical residues with the SLC25A20_CAC to be modelled (35% of identical residues). The TtAAC1 structure is the only available crystallized structure of a mitochondrial carrier in m-conformation on the PDB and it also shares 35% of identical residues with SLC25A20. Residues of the sequence motif of mitochondrial carrier family members were used as reference residues for preparing the sequence structure pairwise alignment employed for generating the 3D comparative models of CAC protein in c-conformation and in m-conformation. During the modeling process, the Cα backbone of the SLC25A20_CAC target was restrained to the backbone of the template structure. Distance constraints were placed between the residues of the salt bridges in the SLC25A20_CAC target. A slow molecular dynamics simulation with an annealing procedure [31,52] was repeated to generate 100 optimized SLC25A20_CAC structures under these restraints and constraints. The structural properties of the SLC25A20_CAC models with the best energy were examined in SPDBV and PyMOL (https://pymol.org/2/; accessed on 20 November 2022), and alternative side-chain rotamers were evaluated where side-chain packing led to gaps between transmembrane α-helices [31,52]. Site-directed mutagenesis of the investigated cysteine residues was performed by using the mutagenesis tool of PyMOL and the built 3D model of the CAC wild type protein to create the 3D model the single mutants CAC_C136S, CAC_C155S, and the 3D model of mutants containing only the cysteine residues C136 (C23V/C58V/C89S/C155V/C283S) or C155 (C23V/C58V/C89S/C136V/C283S). The “super” commands implemented in PyMOL was used to calculate the root mean square deviation (RMSD) between the coordinates of the atoms of the backbone of the generated 3D comparative models and the corresponding atoms of the crystallized structures used as protein templates, as previously described [53].

### 4.7. Docking Analysis

A gridbox consisting of residues within 10 Å from C136 was prepared for investigating the binding of PAT at the level of C136, the cysteine residue making CAC more sensitive to PAT inhibition. The selected gridbox included residues located between the proposed MC regulatory site and the substrate binding region [31,52]. Docking analysis was performed on the RnCAC wild-type protein and on the two RnCAC 3D comparative models hosting, as unique cysteine, only C136 or C155 using Autodock 1.5.6. Our docking protocols have been previously validated through a redocking analysis of the AAC co-crystallized inhibitors [54].

Concerning the docking parameters, the employed gridbox consists of 84, 84, and 66 grid points along the x, y, and z axis, respectively. The gridbox spacing (i.e., the space between two adjacent grid points) was set to 0.275 Å and the xyz coordinates of the grid center were 26.573 (x) 23.194 (y) 11.025 (z). For the docking protocol, the maximum number of energy evaluations was set to 3000000, the maximum number of generations was set to 30,000, rmstol was set to 2.0 Å, and the number of the lowest free energy binding poses to be saved was set to 50. The poses with the lowest predicted free energy were used for the presented analysis.

### 4.8. Sample Preparation for MS Analysis

After the reconstitution procedure, while 600 μL proteoliposomes were incubated with 100 μM Patulin, another 600 μL remained untreated (control). After 60 min incubation at 20 °C, both the samples were passed through the G75 column to eliminate excess PAT. Each reconstitution was added to 10 volumes of acetone (6 mL) and left overnight at −20 °C. The solution was centrifuged 15 min at 10,000× *g*. Acetone was discarded, and the pellet was dried using N_2_. The pellet was solubilized with sample buffer containing SDS 3% and 100 mM DTE and utilized for protein gel electrophoresis.

### 4.9. In-Gel Digestion

The bands of interest were carefully cut from a 1D gel in pieces of 1–1.5 mm size and placed in a siliconized tube. The gel pieces were covered with 200 µL of a solution composed of 200 mM ammonium bicarbonate and acetonitrile (60:40) and incubated at 37 °C for 30 min (step repeated twice) for de-staining. After gel drying, 30 µL (0.6 µg) of a trypsin solution were added alongside 70 µL of 40 mM ammonium bicarbonate containing 9% acetonitrile, and the solution was incubated overnight at 37 °C. Following the incubation, the liquid from the gel was recovered and acidified with 5 µL of formic acid to stop the digestion. The sample was dried under a nitrogen flow up to the half of starting volume.

### 4.10. MALDI-TOF MS Analysis

For MALDI-MS analysis, the tryptic digest was mixed with CClCA (10 mg/mL in 70% acetonitrile and 0.1% TFA) as the matrix at a 1:1 *v*:*v* ratio. Typically, 1 μL of the blend was applied to the metallic sample plate; after drying the sample spot was washed on target twice with ultrapure water. All experiments were performed using a 5800 MALDI-ToF/ToF analyzer (SCIEX, Darmstadt, Germany) equipped with a neodymium-doped yttrium lithium fluoride (Nd:YLF) laser (345 nm) in a reflectron positive ion mode with a mass accuracy of 10 ppm. In both MS and MS/MS modes, 1000 laser shots were typically accumulated by a random rastering pattern, at laser pulse rates of 400 and 1000 Hz, respectively. MS/MS experiments were performed setting a potential difference of 1 kV between the source and the collision cell; ambient air was used as the collision gas with a medium pressure of 10−6 Torr. The delayed extraction time was set at 450 ns. DataExplorer software 4.0 (Sciex) was used to control the acquisitions and to perform the initial elaboration of data, whereas SigmaPlot 12.5 was used to graph the final mass spectra.

### 4.11. RPLC-ESI-MS Instrumentation and Operating Conditions

An Ultimate 3000 UHPLC chromatographic system coupled by a heated electrospray source ionization (HESI) to a Velos Pro linear ion trap (LIT) mass spectrometer (Thermo Scientific, Waltham, MA, USA) was employed. Chromatographic separation was performed at a flow rate of 0.2 mL/min and column temperature of 40 °C using a Phenomenex Aeris WIDEPORE 200 Å C18 column (250 × 2.1 mm, 3.6 µm) equipped with Phenomenex AJO 8783 WIDEPORE C18 (2 × 2.1 mm ID) security guard cartridge with mobile phases H_2_O (A) and ACN (B) both with 0.1% FA in volume. The chromatographic gradient was set as follows: min 0–2 5% B phase isocratic; min 2–12 from 5% to 35% B phase, min 12–17 35% B phase isocratic, min 17–35 from 35% to 100% B phase, min 35–37 100% B phase, min 37–39 from 100% to 5% B phase, min 39–45 5% B phase isocratic. For MS acquisitions, parameters were set as follows: sheath gas flow rate 30 a.u. (arbitrary units); auxiliary gas flow rate 15 a.u.; spray voltage 2.5 kV; capillary temperature 275 °C; S-Lens radio frequency level 60 a.u. Acquisitions were performed in positive ion mode within the *m*/*z* interval 130–2000. Collisional induced ionization (CID) for MS/MS experiments was set at 35% of normalized collision energy. The control of LC-MS instrumentations and the first processing of data was performed by the Xcalibur software 2.2 SP1.48 (Thermo Scientific). Data processing of mass spectra was performed by SigmaPlot 12.5. ProteinProspector (v. 6.2.2) software was used to perform a database search of proteins or peptides.

### 4.12. Database Searching

Peptide mass fingerprinting (PMF) was carried out with Protein Prospector (Regents of the University of California) software by employing the MS-Fit tool. Proteins were identified using SwissProt database with Rattus norvegicus as taxonomy restriction. Search parameters for MS analysis were the following: peptide mass tolerance 100 ppm, allowed trypsin missed cleavages up to 3. No fixed chemical modification was inserted, but the oxidation of methionine residues was considered a variable modification. The acquired MS/MS data set was processed by mMass™ 5.5.0 using MASCOT search engine (Matrix Science, London, UK) and MS-Tag from Protein Prospector. A tolerance of 0.5 Da was set for the precursor ion and MS/MS fragment ions.

### 4.13. Other Methods

The amount of reconstituted protein was estimated from Coomassie blue stained SDS-PAGE gels using the Chemi Doc imaging system equipped with Quantity One Analysis Software (Bio-Rad) as previously described [28].

### 4.14. Statistical Analysis

Statistical analysis was performed using the Student’s *t*-test, as indicated in figure legends. Values of * *p* < 0.01 were considered statistically significant. Data points were derived from means of three different experiments as specified in the figure legends.

## Figures and Tables

**Table 1:** Detectable (S/N ≥ 3) peptides after in-gel trypsin digestion of the 30 kDa band excised from the control and treated samples.

Experimental as [M + H]^+^ *m*/*z*		Theoretical *m*/*z*	Start-End a.a.	Sequence
Control	Treated			
1009.49	1009.52	1009.54	203–211	SVHDLSVPR
1025.49	1025.52	1025.52	259–267	EEGVTSLYK
1025.49	1025.52	1025.54	159–166	LYQEFGIR
1053.56	1053.58	1053.61	293–301	ILNWIAPNL
**1147.57**	**-**	**1147.61**	**136–146**	**CLLQIQASSGK**
1153.58	1153.61	1153.63	158–166	KLYQEFGIR
1208.64	1208.66	1208.69	2–12	AEEPKPISPLK
-	1357.68	1357.73	167–178	GFYKGTALTLMR
1536.78	1536.81	1536.83	255–267	ELIREEGVTSLYK
1579.83	1579.79	1579.80	237–250	FQTAPPGKYPNGFR
-	1667.89	1667.95	220–234	GIFNWVVAIPPDVLK
1937.81	1937.85	1937.88	179–194	DVPASGMYFMTYEWLK
1953.81	1953.84	1953.87	179–194	DVPASGMoxYFMTYEWLK
2313.21	2313.20	2313.24	13–35	NLLAGGFGGVCLVFVGHPLDTVK
-	**2456.23**	2456.26	**136–158**	**CLLQIQASSGKNKYSGTLDCAKK**
2781.34	2781.32	2781.34	171–194	GTALTLMRDVPASGMYFMTYEWLK
3345.58	3345.62	3345.60	103–133	SPEDELTYPQLFTAGMLSGVFTTGIMTPGER
3361.52	3361.54	3361.59	103–133	SPEDELTYPQLFTAGMoxLSGVFTTGIMTPGER
Coverage (%)				
52.5	60.1			

## Data Availability

The data presented in this study are available on request from the corresponding author.

## Supplementary Materials

The following supporting information can be downloaded at: https://www.mdpi.com/article/10.3390/ijms24032228/s1.

## Abbreviations

MC (Mitochondrial Carrier), Patulin (PAT), CAC (carnitine/acylcarnitine carrier), WHO (World Health Organization), 1,4-Dithioerythritol (DTE), MS (mass spectrometry).
