# Supplementary material for: The Mycotoxin Patulin Inhibits the Mitochondrial Carnitine/Acylcarnitine Carrier (SLC25A20) by Interaction with Cys136 Implications for Human Health
Source: Int J Mol Sci. 2023 Jan 23;24(3):2228. doi: 10.3390/ijms24032228 (PMC9917099; doi:10.3390/ijms24032228)
Supplement: Supplementary file 1 [file ijms-24-02228-s001.zip › ijms-2120159-supplementary.pdf]

Supplementary Materials

The mycotoxin patulin inhibits the mitochondrial carnitine/acylcarnitine carrier (SLC25A20) by interaction with Cys136. Implications for human health

Nicola Giangregorio[1,†,*], Annamaria Tonazzi[1,†], Cosima Damiana Calvano[2], Ciro Leonardo Pierri[3], Giovanna Incampo[4], Tommaso R.I. Cataldi[2] and Cesare Indiveri[1,5]

[1] CNR Institute of Biomembranes, Bioenergetics and Molecular Biotechnologies (IBIOM), Via Amendola 122/O, 70126 Bari, Italy
[2] Department of Chemistry, University of Bari Aldo Moro, via Orabona 4, 70126, Bari, Italy
[3] Department of Pharmacy-Pharmaceutical Sciences, University of Bari Aldo Moro, via Orabona 4, 70126, Bari, Italy
[4] Department of Bioscience, Biotechnology and Environment, University of Bari, Italy
[5] Department DiBEST (Biologia, Ecologia, Scienze della Terra) Unit of Biochemistry and Molecular Biotechnology, University of Calabria, Via Bucci 4C, 87036 Arcavacata di Rende, Italy
† These authors contributed equally to this work.
* Correspondence: n.giangregorio@ibiom.cnr.it

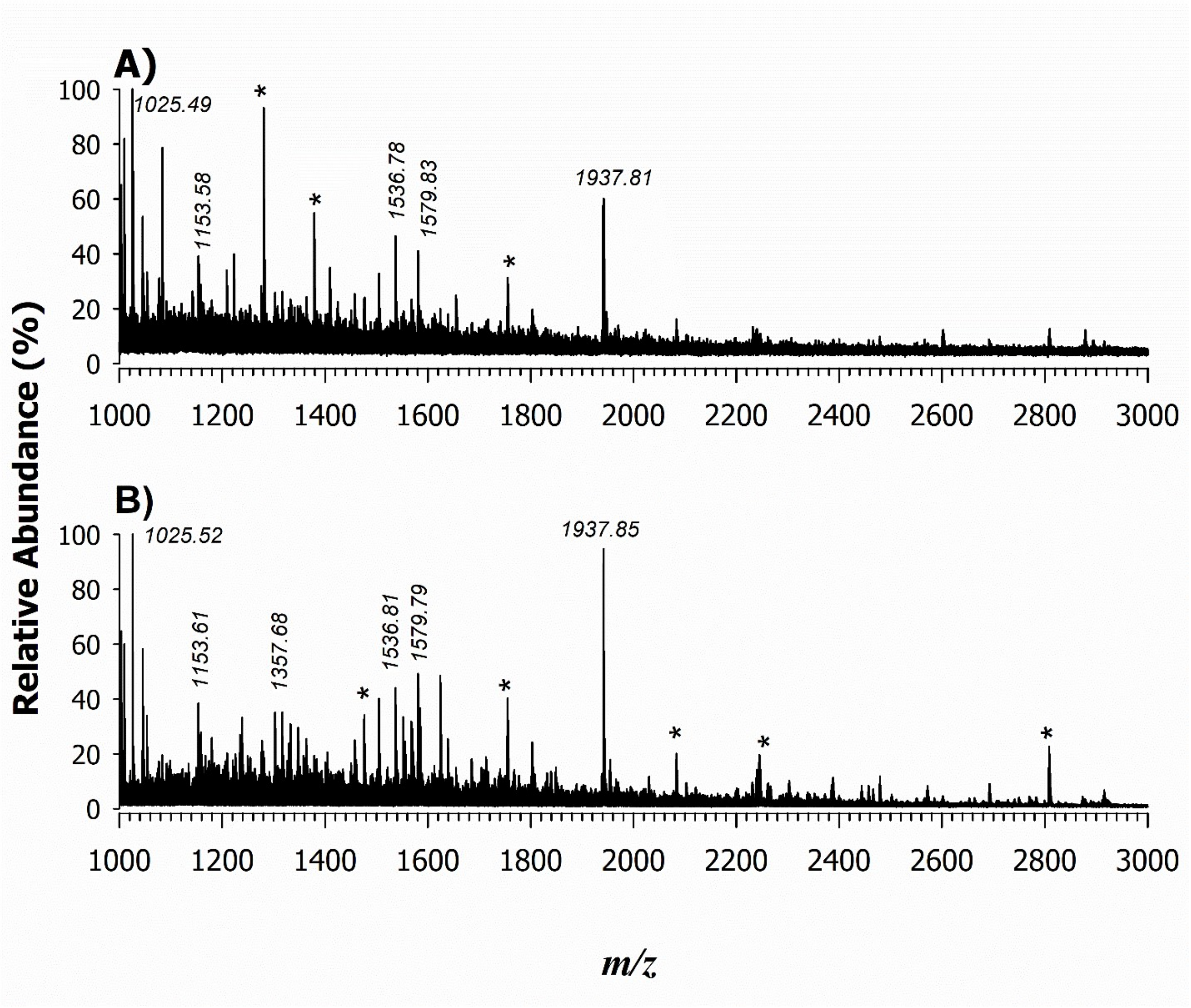

Figure S1. PMF of in-gel digestion of the 30 kDa bands excised from control (A) and (B) PAT-treated samples. Table 1 lists all main peak signals and their amino acid sequence. Asterisked peaks are most likely due to co-extracted CAC and digested proteins

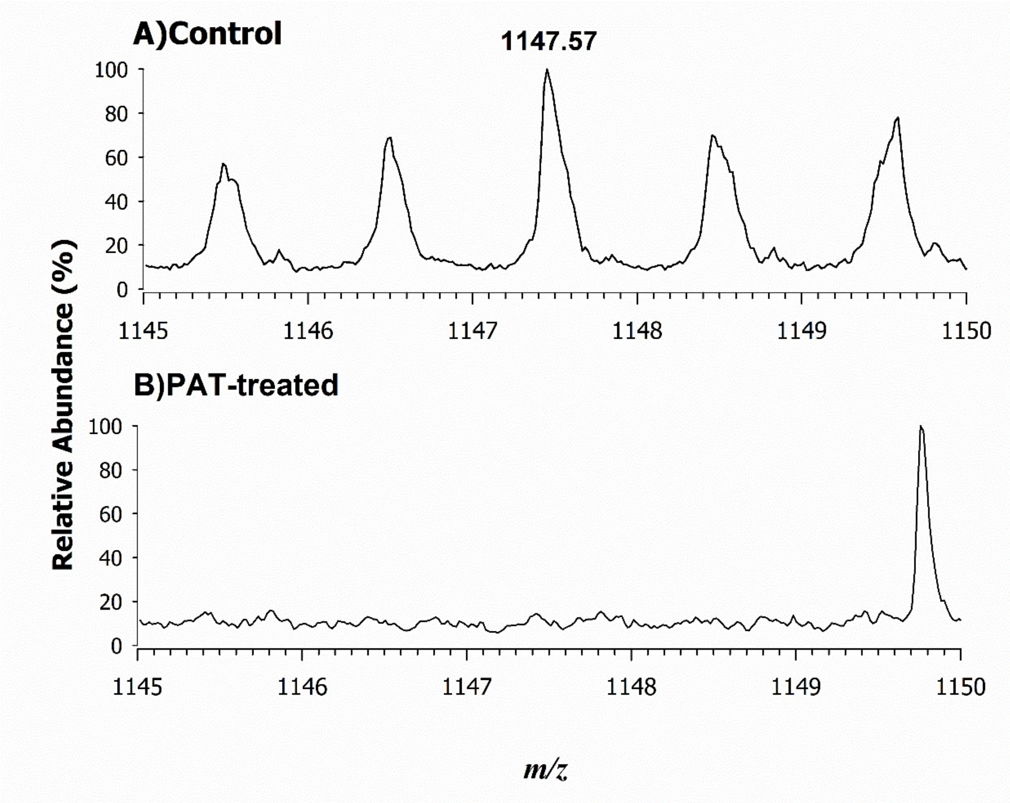

Figure S2. A zoom on the peak assigned as peptide CLLQIQASSGK, at *m/z* 1147.57, is shown for control (A) and PAT-treated (B) samples.

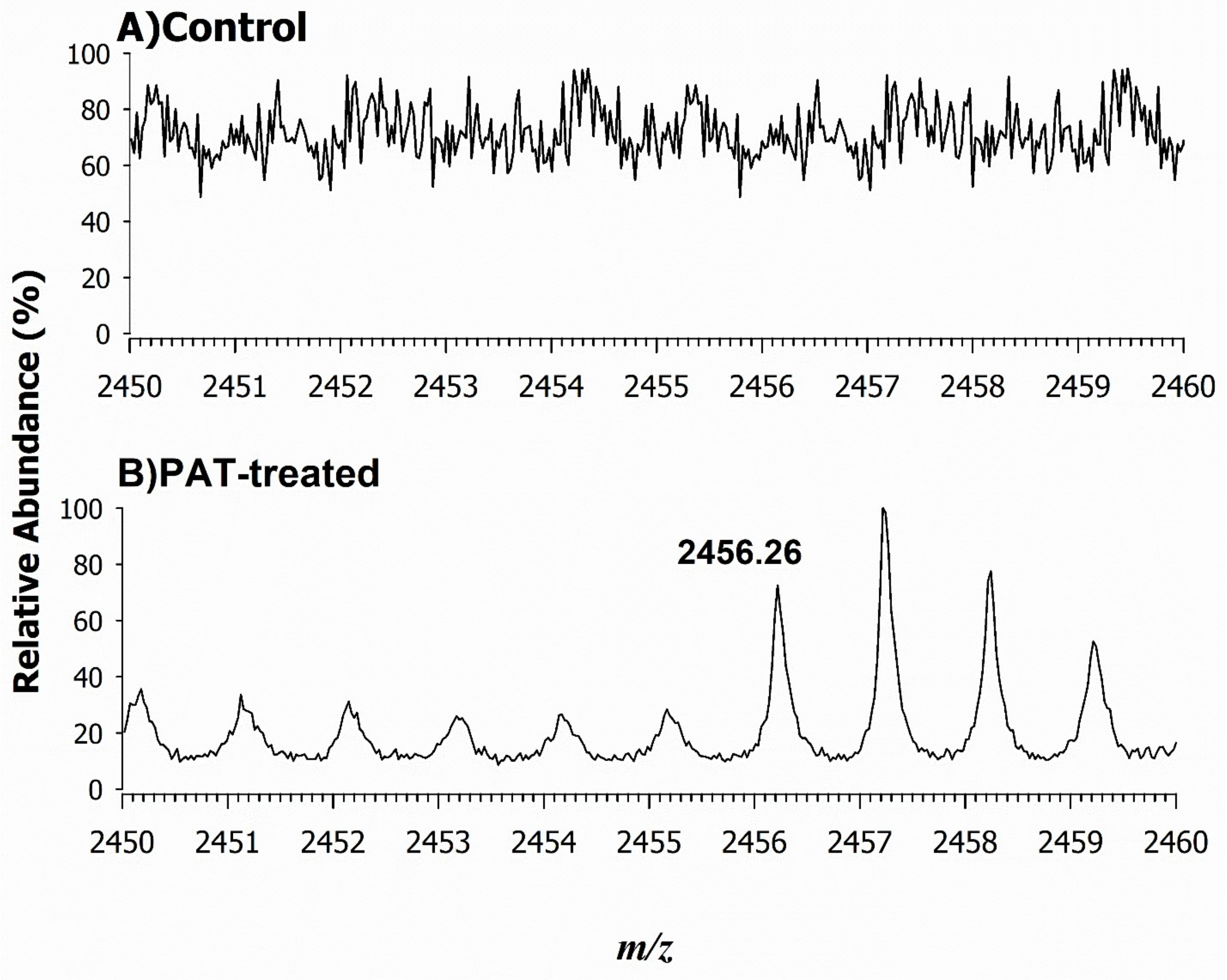

Figure S3. A zoom on the peak assigned as peptide CLLQIQASSGKNKYSGTLDCAKK, at *m/z* 2456.3, is shown for control (A) and PAT-treated (B) samples.

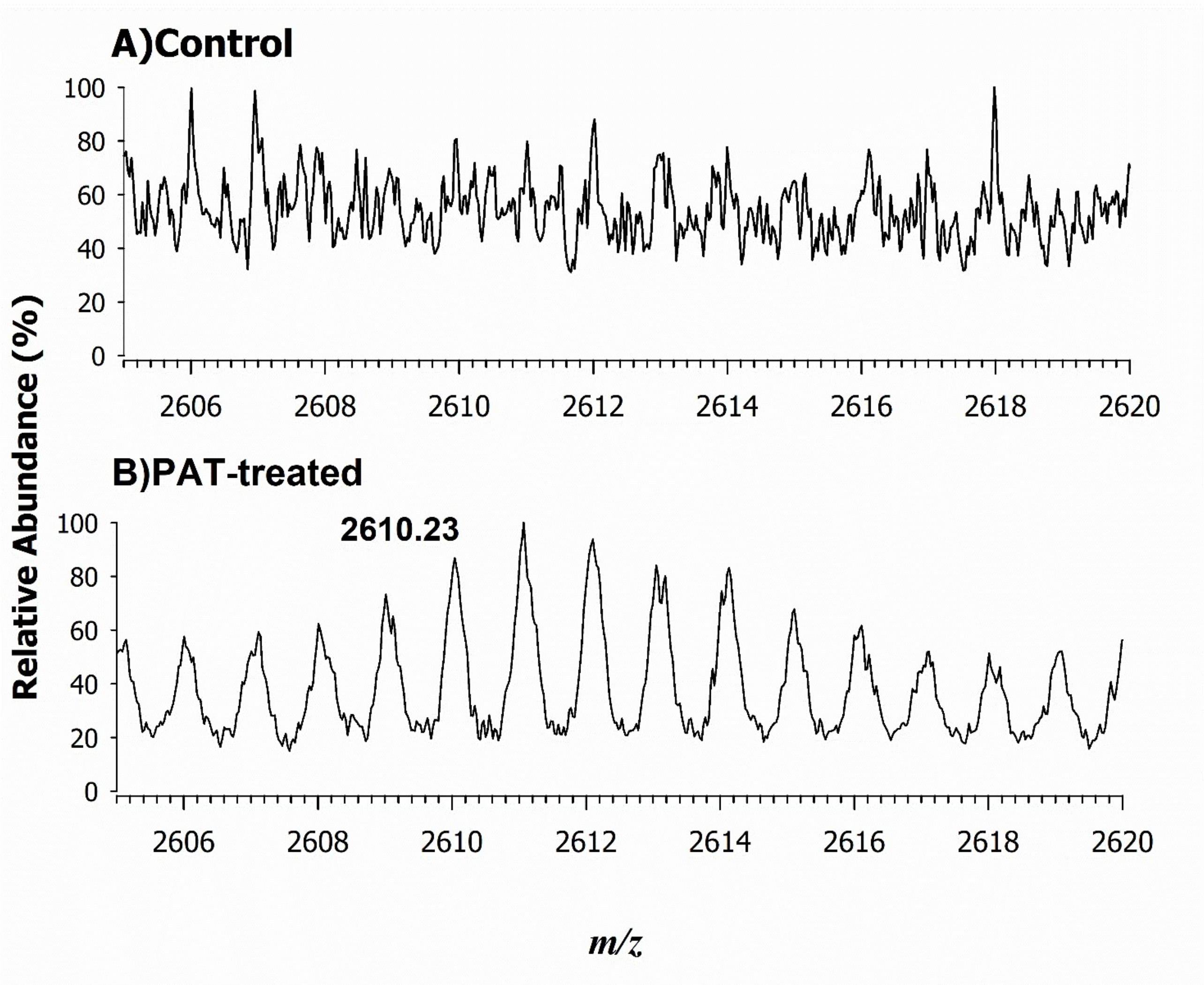

Figure S4. A zoom on the peak associated with modified peptide, at *m/z* 2610.23, is shown for control (A) and PAT-treated (B) samples.
